# SARS-CoV-2 neutralizing antibody bebtelovimab – a systematic scoping review and meta-analysis

**DOI:** 10.3389/fimmu.2023.1100263

**Published:** 2023-08-28

**Authors:** Mabel Nyit Yi Liew, Kok Pim Kua, Shaun Wen Huey Lee, Kon Ken Wong

**Affiliations:** ^1^ Pharmacy Unit, Puchong Health Clinic, Petaling District Health Office, Ministry of Health Malaysia, Petaling, Selangor, Malaysia; ^2^ School of Pharmacy, Monash University, Subang Jaya, Selangor, Malaysia; ^3^ Health and Well-being Cluster, Monash University, Subang Jaya, Selangor, Malaysia; ^4^ Gerontechnology Laboratory, Monash University, Bandar Sunway, Selangor, Malaysia; ^5^ Faculty of Health and Medical Sciences, Taylor’s University, Subang Jaya, Selangor, Malaysia; ^6^ Center for Global Health, Perelman School of Medicine, University of Pennsylvania, Philadelphia, PA, United States; ^7^ Department of Medical Microbiology & Immunology, Faculty of Medicine, Universiti Kebangsaan Malaysia, Kuala Lumpur, Malaysia

**Keywords:** bebtelovimab, monoclonal antibody, SARS-CoV-2, COVID-19, omicron, variant, neutralization, spike protein

## Abstract

**Introduction:**

The COVID-19 pandemic is a major global public health crisis. More than 2 years into the pandemic, effective therapeutic options remain limited due to rapid viral evolution. Stemming from the emergence of multiple variants, several monoclonal antibodies are no longer suitable for clinical use. This scoping review aimed to summarize the preclinical and clinical evidence for bebtelovimab in treating newly emerging SARS-CoV-2 variants.

**Methods:**

We systematically searched five electronic databases (PubMed, CENTRAL, Embase, Global Health, and PsycINFO) from date of inception to September 30, 2022, for studies reporting on the effect of bebtelovimab in SARS-CoV-2 infection, using a combination of search terms around ―bebtelovimab‖, ―LY-CoV1404‖, ―LY3853113‖, and ―coronavirus infection‖. All citations were screened independently by two researchers. Data were extracted and thematically analyzed based on study design by adhering to the stipulated scoping review approaches.

**Results:**

Thirty-nine studies were included, thirty-four non-clinical studies were narratively synthesized, and five clinical studies were meta-analyzed. The non-clinical studies revealed bebtelovimab not only potently neutralized wide-type SARS-CoV-2 and existing variants of concern such as B.1.1.7 (Alpha), B.1.351 (Beta), P.1 (Gamma), and B.1.617.2 (Delta), but also retained appreciable activity against Omicron lineages, including BA.2.75, BA.4, BA.4.6, and BA.5. Unlike other monoclonal antibodies, bebtelovimab was able to bind to epitope of the SARS-CoV-2 S protein by exploiting loop mobility or by minimizing side-chain interactions. Pooled analysis from clinical studies depicted that the rates of hospitalization, ICU admission, and death were similar between bebtelovimab and other COVID-19 therapies. Bebtelovimab was associated with a low incidence of treatment-emergent adverse events.

**Conclusion:**

Preclinical evidence suggests bebtelovimab be a potential treatment for COVID-19 amidst viral evolution. Bebtelovimab has comparable efficacy to other COVID-19 therapies without evident safety concerns.

## 1 Introduction

The COVID-19 pandemic is the most significant global public health crisis of this generation, resulting in a high estimated excess mortality rate across the globe (1). Older adults and individuals with multimorbidity are predominantly vulnerable to the severe clinical course of COVID-19, in-hospital complications, and death (2). While several vaccines have been proven to be highly effective in reducing the incidence of hospitalization and death attributed to numerous causative SARS-CoV-2 variants (3), there has been significant hesitancy among the population with vaccine uptake, thus hampering the attainment of vaccination coverage required for population immunity (4). Furthermore, given the increased risks of COVID-19 infection and severe disease associated with inactivated whole-virus vaccines (5), the widespread use in many countries worldwide, particularly in crowded low- and middle-income countries that bear potentially higher risks of emerging SARS-CoV-2 variants becoming the epicenter for further spread and health care crisis warrants the need of effective therapeutic interventions to prevent severe disease progression, hospitalization, and mortality.

A growing body of evidence shows that monoclonal antibody therapies significantly reduce the risk of hospitalization of COVID-19 when administered early (6). Monoclonal antibodies are the largest class of biologicals for use in clinical practice, comprising a myriad of structures, ranging from small fragments to intact, modified, or unmodified immunoglobulins, all of which possess an antigen-binding domain (7). The emergence and proliferation of SARS-CoV-2 variants have been demonstrated to impair the efficacy of monoclonal antibody therapies due to the occurrence of mutations in the antigenic supersite of N-terminal domain or the ACE2-binding site (receptor-binding motif) of SARS-CoV-2, both major binding targets of the neutralizing monoclonal antibodies (8). To date, five types of anti-SARS-CoV-2 antibody drugs have been developed, namely bebtelovimab, bamlanivimab plus etesevimab, casirivimab plus imdevimab, sotrovimab, and tixagevimab-cilgavimab (9).

Of note, circulating variants of concern in the communities affect the effectiveness of each anti-SARS-CoV-2 monoclonal antibody therapy. The emergence and proliferation of SARS-CoV-2 B.1.1.529 Omicron virus has rendered specific monoclonal antibodies ineffective due to a marked reduction in neutralizing activity (10). A live virus focus reduction neutralization test depicts that combinations of monoclonal antibodies, including bamlanivimab plus etesevimab, casirivimab plus imdevimab, as well as tixagevimab-cilgavimab have neutralizing activity against early strain and the Alpha and Delta variants. Nonetheless, etesevimab plus bamlanivimab exhibits dramatically decreased activity against Gamma variant and exerts no inhibitory effect against Omicron and Beta variants. On the other hand, casirivimab plus imdevimab shows efficacy against Beta and Gamma variants, whilst losing neutralizing activity against Omicron. Tixagevimab-cilgavimab elicits inhibitory activity against Beta, Gamma, and Omicron variants, but the titer of monoclonal antibodies required for a 50% reduction in the number of infectious foci (FRNT_50_ or sometimes also referred to as IC_50_) is 24.8 to 142.9 higher for Omicron than for Beta or Gamma. Likewise, sotrovimab remains to have neutralizing activity against Beta, Gamma, and Omicron variants, but nevertheless, the FRNT_50_ value is 3.7 to 198.2 higher for Omicron than for Beta or Gamma (11).

In another experiment, etesevimab plus bamlanivimab is found to have no neutralizing activity against Omicron/BA.2. Casirivimab plus imdevimab can inhibit Omicron/BA.2, but no neutralizing activity is demonstrated against Omicron/BA.1 or Omicron/BA.1.1. Tixagevimab-cilgavimab retains activity against Omicron/BA.2. Sotrovimab has been depicted to have lower neutralizing activity against Omicron/BA.2 compared to Omicron/BA.1, Omicron/BA.1.1, and the ancestral strain. The FRNT_50_ value of each of these monoclonal antibodies is considerably higher for Omicron/BA.2 in comparison with the ancestral strain and other variants of concern (12).

In view of the global dominance of the Omicron variant and the diminished therapeutic effect against the newly emerged variant, the United States National Institutes of Health (NIH) COVID-19 Treatment Guidelines Panel no longer recommends the use of bamlanivimab plus etesevimab, casirivimab plus imdevimab, or sotrovimab for the treatment of COVID-19. At present, tixagevimab-cilgavimab is shown to be safe and efficacious as pre-exposure prophylaxis and potential treatment for mild to moderate COVID-19 (13). On the other hand, bebtelovimab, being the sole monoclonal antibody that remains effective *in vitro* against all circulating Omicron subvariants (14), is approved by the United States Food and Drug Administration (FDA) and the NIH COVID-19 Treatment Guidelines Panel as a therapeutic option in high-risk patients with COVID-19 (9, 15).

One of the strategies to ascertain the role of bebtelovimab in mild to moderate COVID-19 infection is evidence synthesis using existing literature to inform and design studies of this promising therapy. Recognizing this gap, a scoping review is performed to identify and delineate of the current state of research evidence on the effect of bebtelovimab on COVID-19. The findings of the review will be utilized to inform future research within the theme of human IgG1 monoclonal SARS-CoV-2 antibody and possibly other research groups examining biologic drugs and lay a cornerstone of the foundation for formulating laboratory guidance and clinical tools for biomedical researchers to work on therapeutic options for COVID-19 patients.

## 2 Methods

### 2.1 Overview

We conducted a systematic search to identify the preclinical and clinical evidence concerning the therapeutic effects of bebtelovimab in COVID-19. The scoping review was done in accordance with the Preferred Reporting Items for Systematic Review and Meta-Analysis Extension for Scoping Reviews (PRISMA-ScR) (16) and the Joanna Briggs Institute (JBI) (17). Our aim was to present a rigorous, comprehensive, systematic approach to synthesize the current heterogeneous literature to ascertain gaps in knowledge and provide an effective summary for practitioners and guide researchers across the disciplines ranging from the laboratory bench to real-world clinical environment. The synthesis of evidence focused on *in vitro* studies, *in vivo* studies, clinical trials, and modeling studies that investigated the effect of bebtelovimab on SARS-CoV-2 infection.

### 2.2 Search strategy and selection criteria

We searched five electronic bibliographic databases, namely PubMed, Cochrane Central Register of Controlled Trials (CENTRAL), Embase, Global Health, and PsycINFO, for articles published in English from database inception until September 30, 2022 using a combination of search terms relating to bebtelovimab and COVID-19, as provided in the **Appendix**. Reference lists and tracked citations of retrieved articles were scrutinized to locate relevant publications not detected during the database searches. Preprint servers of bioRxiv and medRxiv were also searched for additional studies. Authors were contacted for further information that was not available in the published material (18).

Publications were deemed eligible for inclusion if they reported on preclinical or clinical findings regarding the use of bebtelovimab in SARS-CoV-2 infection. Studies were excluded if they reported aggregation of outcomes from different monoclonal SARS-CoV-2 antibody therapies but did not evaluate an actual or specific impact of bebtelovimab.

### 2.3 Article selection

All citations were imported into EndNote (version X9) reference management software and duplicates were removed. Study selection was undertaken by two reviewers and occurred in two stages, comprising initial title and abstract screening, followed by full-text review. In each stage, two reviewers independently evaluated each study against a set of pre-specified inclusion and exclusion criteria to determine whether it should move forward. Any incongruences were resolved through discussion, or, in the case of no consensus, a third reviewer was involved.

### 2.4 Data analysis

A standardized data extraction form was developed and independently piloted using Microsoft Word. Data from included studies such as details of therapeutic intervention, study characteristics and design, data for our focal outcomes, analytical methods, results, as well as individual study strengths and limitations were independently extracted by two reviewers. The complete data extraction was verified by a third reviewer. All findings were subsequently collated and summarized through the description of narrative synthesis approach. In light of variability in the study designs, we did not plan to formally appraise the methodological quality of the included studies. However, we did provide comments on the limitations of the studies. We also estimated summary risk ratio (RR) using pairwise random-effects meta-analysis.

## 3 Results

**Figure 1:**
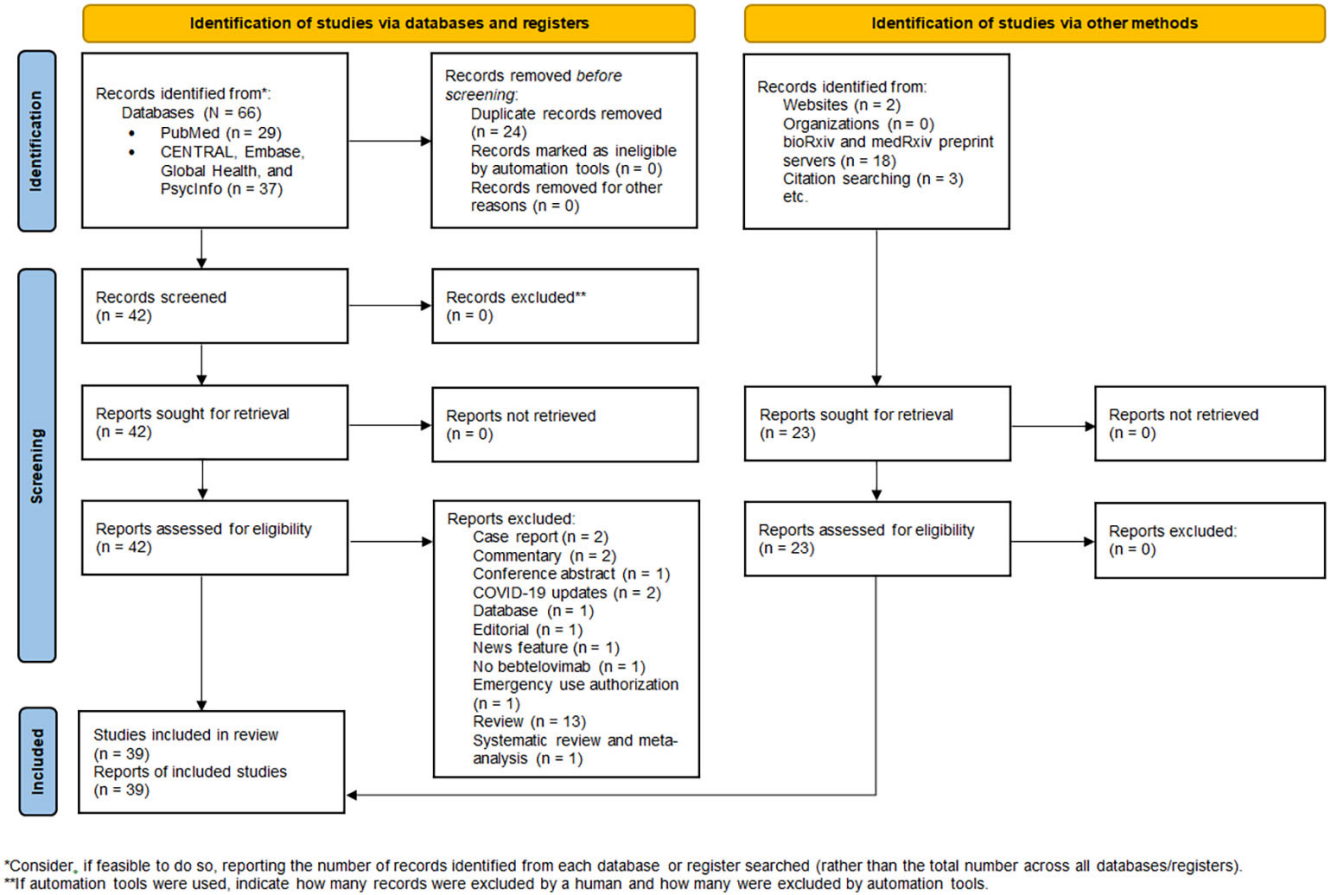
PRISMA 2020 flow diagram for new systematic review which included searches of databases, registers, and other sources.

The database search yielded 66 records, of which 24 duplicate records were removed. 23 additional articles were identified by manual searching. Hence, 65 full-text articles were assessed for eligibility, of which 39 were included in the review (**Figure 1**). 34 studies were non-clinical research (19–52), encompassing *in vitro* virus neutralization experiments (19–50), immunoinformatic analysis (51), and deep mutational scanning (52). The remaining 5 studies were clinical research (18, 53–56), comprising randomized controlled trial (53) and retrospective cohort studies (18, 54–56). 17 studies were conducted in the United States (28, 30, 34, 35, 38, 42, 45, 49, 50, 56), 8 in China (19, 23–26, 31, 48), 7 in Europe (20–22, 29, 36, 37, 41), 4 in Japan (39, 40, 46, 47), 2 in India (32, 51), and 1 across three countries, namely United States, Argentina, and Puerto Rico (53). A summary of the main characteristics of each individual study is outlined in **Tables 1**, **2**.

**Table 1:** Characteristics and results of included non-clinical studies.

Study (year), country	Virus type ^a^	Cell line	Inoculum ^b^	Incubation (hours)	Control	Variants	Main findings
Ai, et al. (2022), China (19)	PV (VSV)	Vero	NA	24	B	BA.1, BA.1.1, BA.2, BA.3	Bebtelovimab maintained its neutralization potency against all Omicron sublineages tested.
Andreano, et al. (2022), Italy (20)	Infectious	Vero	NR	72 – 96	B	BA.1, BA.2, BA.4, BA.5	Bebtelovimab had high neutralization potency against all Omicron sublineages, showing an IC_100_ of 11.1, 15.6, 44.2, and 62.5 ng/ml against Omicron BA.1, BA.2, BA.4, and BA.5 respectively.
Arora, et al. (2022), Germany (21)	PV (VSV)	Vero	NA	16 – 18	B.1	BA.1, BA.2, BA.2.12.1, BA.4, BA.5	Bebtelovimab neutralized all emerging Omicron subvariants tested with similarly high efficacy.
Bruel, et al. (2022), France (22)	Infectious	U2OS-ACE2 GFP1-10 or GFP11	NA	18	B.1.617.2	BA.2, BA.4, BA.5	Bebtelovimab remained fully active against Omicron BA.2, BA.4, and BA.5. Bebtelovimab displayed similar levels of binding and activation of NK-mediated antibody-dependent cellular cytotoxicity against all strains.
Cao, et al. (2022), China (23–26)	PV (VSV)	Huh-7	10^3^	24	B.1	BA.1, BA.1.1, BA.2, BA.2.12.1, BA.2.13, BA.2.74, BA.2.75, BA.2.75.2, BA.2.75.4, BA.2.76, BA.2.77, BA.2.79, BA.3, BA.2.38, BA.2.38.1, BA.4, BA.4.6, BA.5, BA.5.1.12, BA.5.2.7, BA.5.5.1, BA.5.6.2, BF.16, BL.1	Bebtelovimab showed potent neutralizing activity against the majority of assayed Omicron subvariants, except BA.2.38.1, BA.5.2.7, and BA.5.6.2.
Chakraborty, et al. (2022), India (51)	NA	NA	NA	NA	B	BA.1, BA.2, BA.2.12.1, BA.3, BA.4, BA.5	Immunoinformatics simulation depicted L452R/Q498R double mutations in Omicron subvariants caused an approximately 6% reduction in binding affinities of bebtelovimab.
Duerr, et al. (2022), USA (27)	PV (HIV) and Infectious	293T-ACE2 Vero/TMPRSS2	0.2 MOI 100 – 180 PFU	NA	B.1	AY.45, BA.1, BA.2, AY.45-BA.1	Neutralization assays using infectious and pseudotyped viruses depicted bebtelovimab retained activity against all variants tested.
Fan, et al. (2022), USA (28)	PV (HIV)	293T-ACE2	NA	48	B.1	BA.1, BA.2	Bebtelovimab retained at least partial efficacy against Omicron variants by targeting a Class 3 receptor-binding domain epitope adjacent to the BA.1 and BA.2 mutations.
Gruell, et al. (2022), Germany (29)	PV (HIV)	293T-ACE2	NA	48	B.1	BA.2, BA.2.12.1, BA.2.75, BA.4, BA.5	Bebtelovimab demonstrated high BA.2.75 neutralizing potency (IC_50 = _7.0 ng/ml), although the activity was lower than that against the other variants.
Iketani, et al. (2022), USA (30)	PV (VSV)	Vero	NA	12	B.1	BA.1, BA.1.1, BA.2	Bebtelovimab adequately treated all assayed Omicron sublineages, with IC_50_ of approximately 5 ng/ml.
Jian, et al. (2022), China (31)	PV (VSV)	Huh-7	NA	24	B.1	BA.4, BA.4.6, BA.4.7, BA.5, BA.5.9	Bebtelovimab remained potent against R346-mutated BA.4 and BA.5 subvariants.
Kumar, et al. (2022), India (32)	Infectious	Vero/TMPRSS2	1×10^2^ PFU	16 – 40	WA1 isolate	B.1.1.7, B.1.351, P.1, B.1.617.2, BA.1, BA.2	Bebtelovimab showed binding and neutralization potential to Omicron and its sublineages.
Li, et al. (2022), China (33)	Infectious	HEK293F	NA	60	B	BA.1, BA.2, BA.3, BA.4	Bebtelovimab preserved neutralizing activity against all Omicron sublineages tested. None of the four Omicron mutations, namely N440K, G446S, Q498R, and N501Y was found to disrupt the interaction with bebtelovimab, thus indicating its broad neutralizing activity.
Lusvarghi, et al. (2022), USA (34)	PV (HIV)	293T-ACE2-TMPRSS2	1×10^5^− 5×10^5^ RLU	48	B.1	BA.1	Bebtelovimab maintained potency against BA.1 (IC_50 = _3.2 ng/ml) comparable to B.1 (IC_50 = _1.3 ng/ml), whereas antibody cocktail containing bebtelovimab, bamlanivimab, and etesevimab merely retained partial potency (IC_50 = _32.5 ng/ml).
Misasi, et al. (2022), USA (35)	PV (VSV)	293T-ACE2-TMPRSS2	NA	72	B.1	B.1.351, B.1.617.2, BA.1, BA.2, BA.2.12.1, BA.4, BA.5	Bebtelovimab remained active against all variants tested. However, it fully escaped antibody neutralization within two to three rounds of repeated infection *in vitro*.
Sheward, et al. (2022), Sweden (37)	PV (HIV)	293T-ACE2	1×10^5^ RLU	48	B.1	BA.2, BA.2.75, BA.5	Bebtelovimab could neutralize BA.2.75 (IC_50 = _15 ng/ml), but the potency was reduced by 7-fold as compared to B.1 (IC_50 = _2 ng/ml).
Sheward, et al. (2022), Sweden (36)	PV (HIV)	293T-ACE2	1×10^5^ RLU	44 – 48	B.1	BA.2.10.4, BA.2.75.2, BA.4.6, BA.5	Bebtelovimab potently neutralized all emerging Omicron sublineages tested.
Starr, et al. (2022), USA (52)	NA	NA	NA	NA	B	BA.1, BA.2	Deep mutational scanning revealed a broadening of the sites of escape from bebtelovimab binding BA.1 and BA.2 compared to the ancestral strain ascribable to mutations at residues K444, V445, P499, and G446.
Syed, et al. (2022), USA (38)	Infectious	293T-ACE2/ACE2-TMPRSS2 and Vero-E6	50 PFU	72	WA1 isolate	B.1.617.2, B.1.1.529	Bebtelovimab had potent neutralization activity against all variants tested, with IC_50_ of less than 10 ng/ml.
Takashita, et al. (2022), Japan (39)	Infectious	Vero-hACE2- TMPRSS2	1×10^3^ FFU	18	NC002 isolate	BA.1.1, BA.1, BA.2, BA.2.12.1, BA.4, BA.5	Bebtelovimab efficiently neutralized BA.2.12.1, BA.4, and BA.5, with similar IC_50_ values as the ancestral strain.
Takashita, et al. (2022), Japan (40)	Infectious	Vero-hACE2- TMPRSS2	1×10^3^ FFU	18	NC002 isolate	BA.2, BA.2.75, BA.5	Bebtelovimab efficiently neutralized BA.2.75 (IC_50 = _6.21 ng/ml), however, this value was 4.4-fold higher compared to the ancestral strain.
Turelli, et al. (2022), Switzerland (41)	PV (HIV) and Infectious	Vero-E6/Calu-3	3×10^3^ PFU	48	B.1	B.1.1.7, B.1.351, P.1, B.1.617.2, BA.1, BA.2, BA.4, BA.5	Bebtelovimab displayed good action against BA.4 and BA.5, with IC_50_ values of 12 ng/ml and 15 ng/ml respectively. In the Delta variant, spike mutations K444T, V445G, and G446V conferred resistance to bebtelovimab. In the Omicron BA.4 variant, mutations in the spike protein, namely K444T, V445G, and P499H suppressed neutralization activity of bebtelovimab.
Wang, et al. (2022), USA (42)	PV (VSV)	Vero-E6 and HEK293T	NA	24	B	BA.1, BA.1.1, BA.2, BA.2.12.1, BA.4, BA.5	Bebtelovimab retained exquisite *in vitro* potency against BA.2.12.1, BA.4, and BA.5, with IC_50_ below 3 ng/ml.
Wang, et al. (2022), USA (43)	PV (VSV)	Vero-E6 and HEK293T	NA	24	B.1	BA.2, BA.2.12.1, BA.2.75, BA.4, BA.5	Bebtelovimab retained potent neutralizing activity against all Omicron subvariants, with IC_50_ below 10 ng/ml. BA.2.75 demonstrated slight resistance to bebtelovimab, albeit modestly at a 3.7-fold loss in neutralization.
Wang, et al. (2022), USA (44)	PV (VSV)	Vero-E6 and HEK293T	NA	24	B.1	BA.2, BA.4, BA.4.6, BA.4.7, BA.5, BA.5.9, BA.4/5-R346T, BA.4/5-R346S, BA.4/5-N658S	Bebtelovimab retained potent activity against all circulating forms of Omicron subvariants.
Westendorf, et al. (2022), USA (45)	PV (VSV) and Infectious	293T-ACE2/ACE2-TMPRSS2 and Vero-E6	NA	72	B.1	B.1.1.7, B.1.351, P.1, B.1.617.2, B.1.526, B.1.427/B.1.429, BA.1, BA.2	Bebtelovimab bound and potently neutralized all variants tested.
Yamasoba, et al. (2022), Japan (46)	PV (HIV)	HOS-ACE2-TMPRSS2	2 × 10^4^ RLU	48	B.1.1	BA.1, BA.2, B.1.617.2, BA.2.11, BA.2.12.1, BA.4, BA.5	Bebtelovimab was approximately 2 times more effective against BA.2 and all Omicron subvariants tested as compared to wild-type virus.
Yamasoba, et al. (2022), Japan (47)	PV (HIV)	HOS-ACE2-TMPRSS2	2.5 × 10^4^ RLU	48	B.1.1	BA.2, BA.2.75, BA.4, BA.5	Bebtelovimab demonstrated strong antiviral effect against BA.2, BA.4, and BA.5. In comparison, BA.2.75 showed about a 20 to 25-fold resistance to neutralization, suggesting that bebtelovimab may not be a good choice to treat BA.2.75 infection.
Zhang, et al. (2022), China (48)	Infectious	Vero	600 PFU/ml	96	WIV04 isolate	B.1.617.2, BA.1	Bebtelovimab exhibited neutralizing potency against wild-type (IC_50_ = 40.9 ng/ml), B.1.617.2 (IC_50_ = 50.8 ng/ml), and BA.1 (IC_50_ = 17.3 ng/ml).
Zhou, et al. (2022), USA (49)	PV (HIV)	293T-ACE2-TMPRSS2	NA	72	B.1	B.1.1.7, B.1.351, P.1, B.1.617.2, BA.1	Bebtelovimab retained binding and potent neutralization of all variants assessed, including BA.1 and BA.2 sublineages (IC_50_ = 5.1 and 0.6 ng/ml respectively).
Zhou, et al. (2022), USA (50)	PV (HIV)	293T-ACE2	0.2 MOI	48	B.1	B.1.617.2, BA.1, BA.2	Bebtelovimab potently neutralized all variants tested, including BA.1 (IC_50_ = 26.2 ng/ml), BA.2 (IC_50_ = 11.5 ng/ml), and individual point mutated BA.2 viruses (IC_50_ range = 2.8 – 11.7 ng/ml).

a PV, pseudotyped virus; HIV, human immunodeficiency virus; VSV, vesicular stomatitis virus; MLV, murine leukemia virus.

b The preclinical studies reported inoculum as 50% tissue culture infectious doses (TCID_50_), relative light units (RLU), plaque forming units (PFU), focus forming units (FFU), or transducing units (TU). The clinical study reported inoculum as multiplicity of infection (MOI).

**Table 2:** Characteristics and results of included clinical studies.

Study (year), country	Study design	Study population	Age group (years)	Active treatment	Control treatment	Dominant variant	Main findings
Chen, et al. (2022), USA (18)	Retrospective cohort study	Individuals with COVID-19 infection before receiving tixagevimab-cilgavimab (n=121) Individuals with breakthrough COVID- 19 infection following receipt of tixagevimab-cilgavimab (n=102)	Median: 54.5 (Range: 18 – 79) Median: 60.5 (Range: 25 – 99)	Bebtelovimab (n=34), sotrovimab (n=58), casirivimab-imdevimab (n=10), nirmatrelvir-ritonavir (n=46), or remdesivir (n=39)	No treatment (n=36)	BA.1 (prior to tixagevimab-cilgavimab prophylaxis) BA.5 (after tixagevimab-cilgavimab prophylaxis)	Among patients who developed COVID-19 infection prior to tixagevimab-cilgavimab, 36 (29.8%) were hospitalized, including 8 (6.6%) required ICU admission. No COVID-related deaths occurred. Among patients who developed COVID-19 after receiving tixagevimab-cilgavimab, 6 (5.9%) were hospitalized, but none was admitted to ICU. There was no COVID-related mortality. 34 patients (33.3%) received bebtelovimab, of whom only one was hospitalized, with a length of stay of 12 days.
Dougan, et al. (2022), USA, Argentina, and Puerto Rico (53)	Randomized controlled trial	Ambulatory patients presenting with mild-to-moderate COVID-19 within 3 days of laboratory-confirmed diagnosis (n=714)	Median: 35 [low-risk cohort] Median: 48.5 – 52.5 [high-risk cohort]	Intravenous bebtelovimab 175 mg over 6.5 minutes (n=125) [low-risk cohort] Intravenous bebtelovimab 175 mg over 30 seconds (n=100) [high-risk cohort]	Placebo (n=128) or intravenous bebtelovimab 175 mg plus bamlanivimab 700 mg plus etesevimab 1400 mg over 6.5 minutes (n=127) [low-risk cohort] Intravenous bebtelovimab 175 mg plus bamlanivimab 700 mg plus etesevimab 1400 mg over 30 seconds or 6.5 minutes (n=226) [high-risk cohort]	Ancestral strains of SARS-CoV-2 (low-risk cohort) Alpha, gamma, delta, and mu lineages (high-risk cohort)	Among low-risk patients, bebtelovimab monotherapy resulted in a greater viral clearance, a reduction in time to sustained symptom resolution, and a similar rate of treatment-emergent adverse events compared to placebo or combination therapy of bebtelovimab plus bamlanivimab plus etesevimab. The incidence of COVID-19-related hospitalization or all-cause deaths by day 29 were similar between treatment groups. 1 death due to COVID-19 on day 5 was reported in a patient who received combination therapy of bebtelovimab plus bamlanivimab plus etesevimab. Among high-risk patients, there were no treatment comparisons made. The proportion of patients with treatment-emergent adverse events was 14.7% in high-risk patients treated with bebtelovimab or combination therapy. Serious adverse events were reported in 2.1% of high-risk patients, including one death due to cerebrovascular accident.
Razonable, et al. (2022), USA (54)	Retrospective cohort study	High-risk patients with a positive SARS-CoV-2 polymerase chain reaction or antigen test (n=3607)	Median: 66.2 (IQR: 52.5 – 74.7)	Intravenous bebtelovimab 175 mg over 1 minute (n=2833)	Oral nirmatrelvir (150 or 300 mg) plus ritonavir (100 mg) twice daily for a. total of 5 days (n=774)	BA.2	Rates of progression to severe illness and ICU admission were similar between bebtelovimab cohort and nirmatrelvir-ritonavir cohort.
Shertel, et al. (2022), USA (55)	Retrospective cohort study	Solid organ transplant recipients who were treated with Bebtelovimab after being tested positive for COVID-19 (n=25)	Median: 52 ((IQR: 44 – 67)	Bebtelovimab (n=25)	NA	BA.1, BA.2	During 1-month of follow-up period, 2 patients required hospital admission. No cases of acute allograft rejection or death were observed.
Yetmar, et al. (2022), USA (56)	Retrospective cohort study	Solid organ transplant recipients diagnosed with mild-to-moderate COVID-19 (n=361)	Mean: 57.7 ± 14.6	Intravenous bebtelovimab 175 mg over 1 minute (n=92)	Intravenous sotrovimab 500 mg (n=269)	BA.2	Hospitalization rates for COVID-19 were similar between bebtelovimab group and sotrovimab group. 3 patients were admitted to ICU, all of whom received sotrovimab. 4 patients died within 30 days of COVID-19 diagnosis, 2 from each treatment group.

### 3.1 Non-clinical studies

The *in vitro* study conducted by Iketani and co-authors investigated the different therapeutic monoclonal antibodies and found that 17 out of 19 of them had diminished neutralization potency against Omicron BA.2 variant (30). Bebtelovimab demonstrated a consistent and high neutralizing potency against all Omicron subvariants despite the difference in antigenicity displayed. A research by Arora and team also yielded results which echoed the similarly high efficacy of bebtelovimab against all Omicron subvariants (21). Another finding by Westendorf and colleagues suggested that bebtelovimab potently neutralized all documented variants of concern, including the dominant Omicron variant and its sublineages circulating globally. The study reported that the bebtelovimab Fab fragment bound to the S protein of the D614G variant with high affinity, with no loss of binding potency to variants of concern such as B.1.1.7 (Alpha) and B.1.351 (Beta), as well as all tested SARS-CoV-2 viruses that had mutations in the N-terminal domain, receptor-binding domain, and the receptor-binding motif (45). Pseudotyped virus neutralization assay confirmed that bebtelovimab retained effect against Alpha, Beta, Gamma, Delta, Epsilon, Delta-Omicron recombinant, and Omicron sublineages, including BA.1.1, BA.2.12.1, BA.2.75, BA.4.6, BA.4.7, and BA.5.9 (19, 21, 23–31, 34–37, 41–47, 49, 50). Likewise, positive results were observed in live virus neutralization assay (**Supplementary Table 1**) (20, 22, 27, 32, 33, 38–41, 45, 48). Bebtelovimab was the only monoclonal antibody that exhibited good potency against most Omicron variants, except BA.2.38.1, BA.5.2.7, and BA.5.6.2 (23).

Structurally, bebtelovimab bound to the receptor-binding domain epitope on the S protein of SARS-CoV-2 that was less inclined to mutations (19, 45). Bebtelovimab was minimally impacted by the mutational changes in Omicron variants (28, 49, 50). Docking of bebtelovimab onto Omicron’s receptor-binding domain detected four amino acid substitutions at the edge of its epitope. Bebtelovimab had minimal side-chain interactions with 3 of the residues (i.e. K440, R498, and Y501) and the loop containing S446 (fourth residue) had conformational flexibility that could facilitate binding of bebtelovimab to the viral spike protein (49). Furthermore, mutations in the Omicron (i.e. N440K, G446S, Q498R, and N501Y) did not affect the interaction with bebtelovimab. Amino acid residues of BA.2 (i.e. Lys440 and Arg498) were found to form H-bonds with Tyr35 and Thr96 of bebtelovimab, whereas a common mutation in BA.1 and BA.3 (i.e. G446S) might cause interaction between Ser446 and Arg60 of heavy chain in bebtelovimab (33). Contrariwise, L452R/Q498R double mutations in Omicron variants could result in an approximately 6% decrease in binding affinities for bebtelovimab (51). A broadening of sites of escape from binding by bebtelovimab were also detected in Omicron BA.1 and BA.2 attributable to mutations at residues K444, V445, P499 and G446, indicating a lower binding affinity of bebtelovimab for Omicron (52).

Bebtelovimab antibody cocktail did not result in an increased potency or synergistic effect against Omicron (49). Complementary findings from an experiment led by Lusvarghi demonstrated bebtelovimab’s potency against Omicron BA.1 comparable to B.1, while antibody cocktail containing bebtelovimab, bamlanivimab, and etesevimab merely retained partial potency (34).

### 3.2 Clinical studies

A randomized clinical trial evaluated the safety and efficacy of bebtelovimab in COVID-19 patients. In the Phase 1 part of the study, Dougan and co-investigators examined ascending doses and infusion rates of intravenous administration of bebtelovimab in 40 patients with low risk of developing severe COVID-19. Pharmacokinetics and pharmacodynamics modeling determined that target therapeutic doses of bebtelovimab 175 mg, bamlanivimab 700 mg, and etesevimab 1400 mg would result in a drug concentration for optimal viral load reduction (53).

Phase 2 of the study examined 380 patients at low risk for severe COVID-19 randomized 1:1:1 to placebo, bebtelovimab 175 mg, or combination therapy of bebtelovimab 175 mg, bamlanivimab 700 mg, and etesevimab 1400 mg, with another 150 high-risk patients randomized 2:1 to bebtelovimab 175 mg or combination therapy of bebtelovimab, bamlanivimab, and etesevimab. An additional treatment arm allocated combination therapy to 176 patients based on the Centers for Disease Control and Prevention (CDC) updated criteria for high-risk. Viral dynamic modeling depicted no discernable difference in viral load reduction between bebtelovimab monotherapy or in combination with bamlanivimab and etesevimab. A simulation developed from the trial demonstrated that older patients over 70 years of age benefited more from the administration of bebtelovimab monotherapy in view of a larger decline from baseline in the viral load. In terms of efficacy, bebtelovimab and combination therapy arms had a lower proportion of patients with persistently high viral load at Day 7 but did not reach statistical significance (p = 0.097 for bebtelovimab versus placebo; p = 0.132 for bebtelovimab plus bamlanivimab plus etesevimab versus placebo). A marked reduction in viral load from baseline to Day 11 was shown in patients in bebtelovimab (p = 0.006) and combination therapy (p = 0.043) groups compared to placebo. The median time to resolution of symptoms was two days shorter with bebtelovimab monotherapy than with placebo (p = 0.003). The incidence of COVID-19-related hospitalization and all-cause mortality by day 29 were similar across treatment groups (1.6% for bebtelovimab; 2.4% for bebtelovimab plus bamlanivimab plus etesevimab; 1.6% for placebo). In high-risk patients, there were no significant differences in viral load, symptom resolution, COVID-19 hospital admission, and mortality among two groups of patients treated with bebtelovimab alone or in conjunction with bamlanivimab and etesevimab (53).

Post-treatment follow-up assessments were carried out in both parts of the trial. Phase 1 identified no reports of COVID-19-related hospitalizations or mortality and increasing doses and infusion rates of bebtelovimab were not correlated with higher rates of treatment-emergent adverse events through at least 24 to 48 hours. No deaths, severe adverse events, or treatment discontinuations occurred. In Phase 2, no discontinuations were ascribed to treatment-emergent adverse events among low-risk patients. The majority of adverse events were mild or moderate, and there was no significant between-group difference in the overall rates (8.8% for bebtelovimab; 12.6% for bebtelovimab plus bamlanivimab plus etesevimab; 7.8% for placebo). In high-risk patients, only one serious adverse event (cerebrovascular accident) resulted in death among recipients of bebtelovimab monotherapy. Similarly, the majority of adverse events were mild or moderate, with overall rates that did not differ significantly between groups (20.0% for bebtelovimab; 16.0% for bebtelovimab plus bamlanivimab plus etesevimab; 11.4% for bebtelovimab plus bamlanivimab plus etesevimab in CDC expanded criteria patients). Two patients who received combination therapy had infusion-related reactions that resolved upon treatment withdrawal, whereas no anaphylactic reaction occurred among patients receiving bebtelovimab alone (53).

A further live virus neutralization assay in the trial depicted combination therapy of bebtelovimab and bamlanivimab had negligible or no neutralizing activity against Omicron variant (IC_99_ > 10,000 ng/ml), while bebtelovimab monotherapy neutralized Omicron variant with a IC_99_ value of less than 2.44 ng/ml, indicating a comparable or greater potency as that of Delta and WA1 isolates (53).

Two retrospective cohort studies of solid organ transplant patients showed bebtelovimab maintained activity against Omicron BA.1 or BA.2 subvariants (55, 56). The rates of hospitalization, intensive care unit admission, and mortality were similar between bebtelovimab and sotrovimab cohorts (56). Shertel and co-workers reported that only 2 of 25 (8.0%) bebtelovimab-treated patients required hospitalization, of whom one needed remdesivir plus dexamethasone therapy due to worsening oxygenation and another experienced obstructive uropathy and acute kidney injury without any symptoms of upper or lower respiratory tract infection. No deaths and acute allograft rejection were observed during the follow-up (55).

Another retrospective cohort study demonstrated that patients who were given bebtelovimab treatment were significantly older and had more underlying comorbidities than those receiving nirmatrelvir-ritonavir. Notwithstanding the increased risk, bebtelovimab cohort showed similar rates of progression to severe disease, ICU admission, and mortality compared to nirmatrelvir-ritonavir cohort (54). Moreover, Chen and colleagues found patients who contracted COVID-19 following tixagevimab-cilgavimab prophylaxis were less likely to require hospital admission than those without prophylaxis. Only 1 of 34 (2.9%) bebtelovimab-treated patients was hospitalized, and none ended in ICU or death (18).

**Figure 2:**
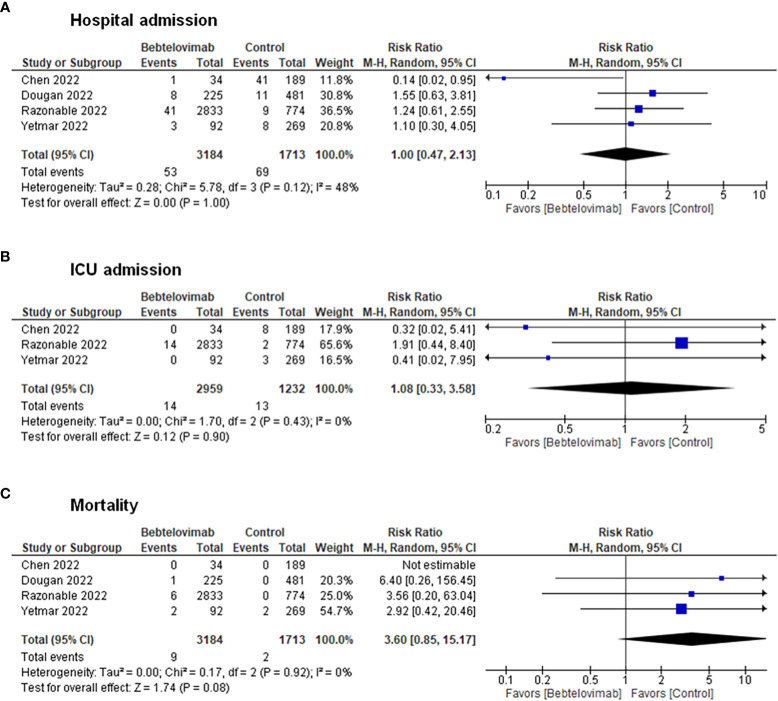
Effects of bebtelovimab compared to control on **(A)** COVID-19-related hospital admission, **(B)** intensive care unit admission, and **(C)** mortality.

Pooling of results from the clinical studies depicted no discernable differences in terms of hospital admissions (RR: 1.00, 95% CI: 0.47 – 2.13, p = 1.00), ICU admissions (RR: 1.08, 95% CI: 0.33 – 3.58, p = 0.90), and death (RR: 3.60, 95% CI: 0.85 – 15.17, p = 0.08) between patients receiving bebtelovimab and patients receiving other COVID-19 therapies (**Figure 2**). Inspection of the funnel plots noted some degree of asymmetry for the three clinical outcomes, suggesting the presence of small-study effects and publication bias (**Supplementary Figure 1**).

## 4 Discussion

The rapid evolution of the SARS-CoV-2 virus continues to challenge our global effort to curb the COVID-19 pandemic. Several clinically available monoclonal antibodies, such as bamlanivimab plus etesevimab, casirivimab plus imdevimab, and sotrovimab are no longer recommended as the treatment for COVID-19 due to a lack of effectiveness against the widely circulating Omicron subvariants. Up till November 2022, bebtelovimab is the sole monoclonal antibody authorized as a treatment for mild to moderate COVID-19 in non-hospitalized patients (9). Our evidence synthesis highlights the therapeutic role of bebtelovimab in COVID-19 infection based on preclinical data depicting its retained potent neutralization against all currently known variants of concern (VOC), along with studies that have demonstrated its clinical safety and efficacy in association with a greater viral clearance and shorter period for symptom resolution. The rate and severity of treatment-emergent adverse events resulting from the use of bebtelovimab are evidenced to be similar to those of placebo and existing monoclonal antibodies in treating both low-risk and high-risk patients. Meta-analyses of clinical studies show no significant differences in risks of COVID-19 hospitalization, ICU admission, or death between patients treated with bebtelovimab and other COVID-19 therapies.

Bebtelovimab, a fully human immunoglobulin G1 (IgG1) monoclonal SARS-CoV-2 antibody, works by targeting the SARS-CoV-2 spike (S) protein’s receptor-binding domain, thereby hindering the spike protein interaction with ACE2 and subsequent viral entry into host cells (45). The *in vitro* efficacy of bebtelovimab is conferred by its ability to bind to an epitope of the SARS-CoV-2 S protein with amino acids that are rarely mutated, as documented in the Global Initiative on Sharing All Influenza Data (GISAID) EpiCoV database (45, 49). Bebtelovimab overcomes mutation-induced structural alterations of the COVID-19 variants by exploiting loop mobility and by minimizing side-chain interactions (49). Overall, there are also consistent findings from clinical studies that demonstrate the effectiveness of bebtelovimab for the treatment of patients infected with SARS-CoV-2 variants (18, 53–56). The collated significant data of this review, in the context of laboratory research and clinical trials, indicate that bebtelovimab is a promising therapeutic option against COVID-19 and newly emerging Omicron sublineages. Our results broadly concur with a recent prediction analysis that bebtelovimab can maintain detectable *in vitro* neutralization against Omicron subvariants such as BA.1, BA.2, BA.4, and BA.5, as well as have a 70.1% (95% CI: 61.9 – 76.8, p < 0.0001) therapeutic efficacy when administered to ambulant COVID-19 positive individuals in preventing illness progression to hospitalization (57).

Monoclonal antibodies have propelled to the forefront in the investigations of pharmacological approaches to treating COVID-19 infection as they are the only appropriate options for clinical use in pediatric patients. Several existing anti-SARS-CoV-2 monoclonal antibodies have been reported to be well-tolerated and raise no safety concerns in children of age between 24 days and 18 years old (58). Whilst bebtelovimab is approved for use in non-hospitalized patients aged 12 years or older, the therapeutic decision to use it across all age pediatric groups should be individualized by incorporating risk factors of progression to severe COVID-19 in the risk-benefit judgment (59). Its indication for a broad population of patients across age groups renders it to be a potential therapeutic strategy to vaccinations and other COVID-19 therapies, especially among those who have underlying immunocompromising condition or multimorbidity, have intolerable adverse effects to COVID-19 vaccination, or are not yet eligible for COVID-19 vaccination.

In tandem with the appearance of multi-mutational SARS-CoV-2 variants such as Delta and Omicron lineages, it is important to enhance the efficacy of bebtelovimab and other potential monoclonal antibodies to overcome new variants that evade natural immunity responses (60). During the period of Delta variant predominance, an existing neutralizing monoclonal antibody sotrovimab resulted in 89% reduction in all-cause mortality and 63% in hospitalization at 28 days compared to untreated patients (61). However, during the period in which Omicron BA.2 was the dominant variant, individuals receiving sotrovimab were associated with higher rates of progression to severe, critical, or fatal COVID-19 (62). Collectively, these real-world findings stand in concurrence with *in vitro* evidence that sotrovimab potently neutralized Omicron B.1.1.529 and BA.1 variants but had low neutralizing activity against Omicron BA.2 and its sublineages (12, 30). Concerning bebtelovimab, the best *in vitro* and clinical data available at present highlight its substantial neutralizing activity against all known SARS-CoV-2 variants, including the Omicron and its new subvariants such as BA.2.75, BA.4, and BA.5, and patients administering bebtelovimab have shown a faster decay in virus titer than placebo. The time frames for clinical studies included in this review comprise pre-Omicron era (53) and Omicron variant (BA.1, BA.2, and BA.5) predominance period (18, 54–56). We could reasonably anticipate that the data carry prominent clinical implications for curbing severe COVID-19 illnesses arising from the current sublineages of the Omicron variant and are likely to resonate with growing evidence from future large-scale randomized controlled trials and real-world studies to recommend the use of bebtelovimab in a broader range of patients. Whilst bebtelovimab appears to be well tolerated in our review, case reports have documented that a patient experienced sinus bradycardia-mediated cardiac arrest immediately following infusion of bebtelovimab (63) and another patient developed colitis 10 days after the use of bebtelovimab (64). Post-marketing surveillance for adverse events and *ad hoc* safety studies are henceforth crucial for earlier detection of safety issues and preventing patients from unnecessary harm (65). Besides, continued laboratory investigations are critical to develop anti-SARS-CoV-2 monoclonal antibodies with better efficacy, safety, and developability features (66). Albeit dedicated wet-lab preclinical research is warranted, this gap can be addressed more rapidly by adding a bioengineering and viral molecular evolution lens to existing lines of research. Instead of just combining different neutralizing monoclonal antibodies, targeting mutated S protein with multivalent nanobody conjugates that can precisely display neutralizing antibodies against SARS-CoV-2 variants has been suggested to have the potential for enhancing the antiviral efficacy (67). However, a recent retrospective cohort study revealed evidence of lack of treatment efficacy among patients infected with SARS-CoV-2 Omicron BA.2, BA.2.12.1, and BA.5 subvariants (68). Hence, well-designed real-world evidence observational studies are important to confirm the efficacy and usage of bebtelovimab. Of note, a next-generation monoclonal antibody may play a pivotal role in inducing rapid immunomodulation and limiting the course of illness, for instance, in debilitating multisystem inflammatory syndrome in children associated with COVID-19 considering the vast potential for improved outcomes with the use of single or combination immunotherapies (69, 70).

Key strengths of our study encompass adherence to scoping review methods, comprehensive search strategy, and inclusion criteria without restrictions on publication status. Limitations of our review are the inclusion of articles published in English only. Supplementary preclinical research is needed to develop neutralizing monoclonal antibodies with optimized clinical efficacy against the evolving variants. The evidence synthesized by this review and the gaps in knowledge reveal that future clinical studies are necessary to foster a deeper understanding of the safety and efficacy of bebtelovimab across different age groups or clinical characteristics, particularly pediatric population and persons with multiple high-risk conditions or comorbidities, the optimal time to initiate treatment, the impact of bebtelovimab on clinical outcomes among patients having previously immunized with different vaccine types or heterologous vaccination regimens, and how Immunocompromised individuals would benefit from additional doses of bebtelovimab in the event of COVID-19 breakthrough infection. The clinical trial included in our review was limited by the exclusive geographical enrollment of patients in North and Latin America, collection of placebo-controlled data among patients at low risk for severe COVID-19, lack of power to assess improvements in clinical outcomes among patients with active treatment before the emergence of Omicron subvariants, use of viral surrogate markers in low-risk younger or healthier subjects for efficacy evaluation, and absence of patient-level clinical data to determine the efficacy of bebtelovimab in patients with symptomatic Omicron infection (53). The retrospective cohort studies had inherent limitations, such as inability to account for sources of residual confounding and selection bias, absence of an untreated control group, and the potential of misclassification bias resulting from administrative data ascertainment, variation in completeness of documentation, inclusion of patients solely in the United States, and lack of laboratory values and biomarkers to better characterize the disease severity. Therefore, further large multinational clinical studies are warranted to resolve these limitations, increase generalizability and evaluate the clinical efficacy and safety of bebtelovimab in diverse patient populations.

## 5 Conclusion

The currently available evidence supports the clinical use of bebtelovimab for patients with SARS-CoV-2 infection who are at increased risk of progression to severe illnesses. With relatively similar pharmacological properties as other previously approved anti-SARS-CoV-2 monoclonal antibodies, bebtelovimab possesses superiority in terms of the ability to neutralize presently circulating Omicron subvariants and different variants of interest. The favorable preclinical and clinical results justify its potential to reserve an active therapeutic role despite the evolutionary trajectories of SARS-CoV-2.

## Data availability statement

The original contributions presented in the study are included in the article/**Supplementary Material**. Further inquiries can be directed to the corresponding author.

## Author contributions

ML – Article selection, data analysis, data interpretation, validation, and writing the original draft. KK – Literature search, study design, article selection, data analysis, data interpretation, validation, and writing the original draft. SL – Literature search, study design, and writing the review & editing. KW – Conceptualization, provision of funding for open-access publishing, project administration, resources, and review. All authors contributed to the article and approved the submitted version.

## Appendix

### Search strategy

#### PubMed, CENTRAL, Embase, Global Health, and PsycInfo

(2019 corona virus) OR (2019 coronavirus) OR (2019 CoV) OR (2019CoV) OR (2019nCoV) OR (2019-nCoV) OR (betacoronavirus) OR (betacoronavir*) OR (corona virus disease 2019) OR (corona virus*) OR (coronavir*) OR (coronavirus disease 2019) OR (coronavirus infection) OR (coronavirus infections) OR (cov 19) OR (CoV 2) OR (Cov19) OR (CoV2) OR (COVID 19) OR (COVID 2019) OR (COVID19) OR (COVID-19) OR (COVID2019) OR (COVID-2019) OR (nCoV) OR (new corona virus) OR (new coronavirus) OR (novel corona virus) OR (novel coronavir*) OR (novel coronavirus) OR (novel CoV) OR (respiratory distress syndrome) OR (sars virus) OR (sars-coronavirus-2) OR (sarscov2) OR (SARSCoV2) OR (SARS-CoV2) OR (SARS-CoV-2) OR (SARS-CoV-2 variant) OR (SARS-CoV-2 variants) OR (severe acute respiratory syndrome) OR (severe acute respiratory syndrome coronavirus 2) AND (bebtelovimab) OR (LY-CoV1404) OR (LY3853113)Preprint servers of bioRxiv and medRxiv (bebtelovimab) OR (LY-CoV1404) OR (LY3853113).
