# Supplementary material for: SARS-CoV-2 neutralizing antibody bebtelovimab – a systematic scoping review and meta-analysis
Source: Front Immunol. 2023 Aug 28;14:1100263. doi: 10.3389/fimmu.2023.1100263 (PMC10494534; doi:10.3389/fimmu.2023.1100263)
Supplement: Supplementary file 1 [file DataSheet_1.docx]

***Supplementary Material***

**Supplementary Figures and Tables**

**Supplementary Figures**

**Supplementary Figure 1.** Funnel plots of the meta-analysis for outcomes of (a) COVID-19-related hospital admission, (b) intensive care unit admission, and (c) mortality.

1. Hospital admission

**
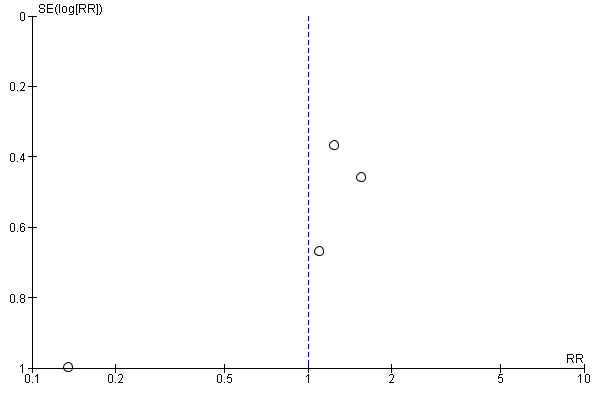
**

1. ICU admission

**
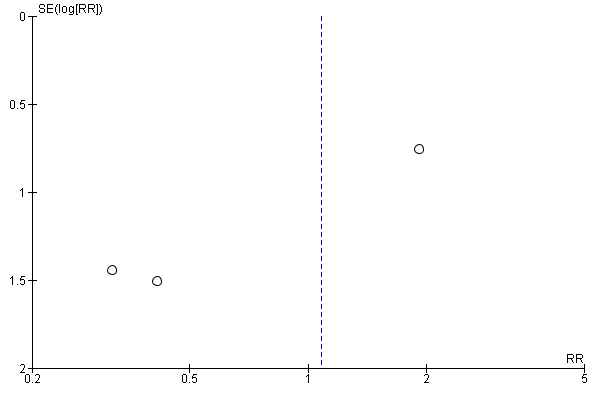
**

1. Mortality

**
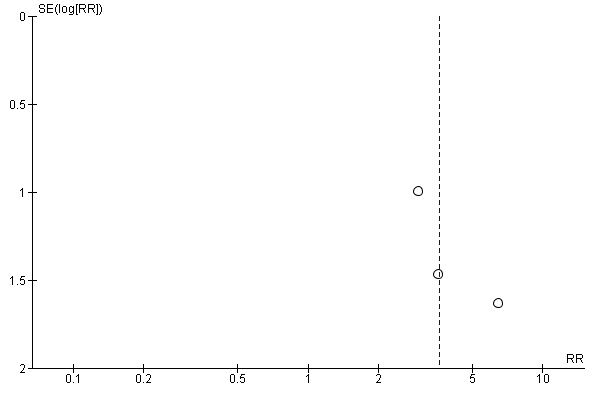
**

**Supplementary Table 1: Neutralization activity of bebtelovimab against SARS-CoV-2 variants reported in included studies.**

|  | Ai, et al. ^19^ ^‡^ | Arora, et al. ^21^ | Duerr, et al. ^27^ | Cao, et al. ^23-26^ | Dougan, et al. ^53^ ᶲ | Gruell, et al. ^29^ | Iketani, et al. ^30^ ^‡^ | Jian, et al. ^31^ | Lusvarghi, et al. ^34^ | Misasi, et al. ^35^ | Sheward, et al. ^37^ | Sheward, et al. ^36^ | Syed, et al. ^38^ | Takashita, et al. ^39^ | Takashita, et al. ^40^ | Turelli, et al. ^41^ | Wang, et  al. ^42^ | Wang, et al. ^44^ | Westendorf, et al. ^45^ | Yamasoba, et al. ^46^ | Yamasoba, et al. ^47^ | Zhang, et  al. ^48^ | Zhou et al. ^49^ | Zhou et al. ^50^ |
| --- | --- | --- | --- | --- | --- | --- | --- | --- | --- | --- | --- | --- | --- | --- | --- | --- | --- | --- | --- | --- | --- | --- | --- | --- |
| Wuhan-01 | - | - | - | - | - | <5.0 | - | - | - | - | - | - | - | - | - | - | - | - | 3.0 | - | - | 40.9 | - | - |
| D614G (B.1) | - | 3.7 | 5.5 | 0.7 | - | 1.0 | - | - | 1.3 | 6.8 | 2.0 | 4.0 | - | - | - | - | 1.0 | 1.5 | - | - | - | - | 3.0 | 9.5 |
| Pangolin-GD | - | - | - | 8.6 | - | - | - | - | - | - | - | - | - | - | - | - | - | - | - | - | - | - | - | - |
| WA1 isolate | - | - | - | - | 3.7 | - | - | - | - | - | - | - | <10.0 | - | - | - | - | - | - | - | - | - | - | - |
| NC002 isolate | - | - | - | - | - | - | - | - | - | - | - | - | - | 2.5 | 1.4 ± 0.8 | - | - | - | - | - | - | - | - | - |
| Alpha (B.1.1.7 and Q lineages) | - | - | - | - | - | - | - | - | - | - | - | - | - | - | - | - | - | - | 1.0 | - | - | - | 30.6 | - |
| Beta (B.1.351 and descendent lineages) | - | - | - | - | - | - | - | - | - | 16.2 | - | - | - | - | - | - | - | - | 2.0 | - | - | - | 4.1 | - |
| Gamma (P.1 and descendent lineages) | - | - | - | - | - | - | - | - | - | - | - | - | - | - | - | - | - | - | 2.0 | - | - | - | 11.5 | - |
| Delta (B.1.617.2 and AY lineages) | - | - | 16.0 | - | 4.9 | - | - | - | - | 22.1 | - | - | <10.0 | - | - | - | - | - | 1.0 | - | - | 50.7 | 3.7 | 9.4 |
| Epsilon (B.1.427 and B.1.429) | - | - | - | - | - | - | - | - | - | - | - | - | - | - | - | - | - | - | 2.0 | - | - | - | - | - |
| Iota (B.1.526 with E484K or S477N) | - | - | - | - | - | - | - | - | - | - | - | - | - | - | - | - | - | - | 2.0 | - | - | - | - | - |
| Kappa (B.1.617.1) | - | - | - | - | - | - | - | - | - | - | - | - | - | - | - | - | - | - | 2.0 | - | - | - | - | - |
| Omicron  (B.1.1.529) | - | - | - | - | - | - | - | - | - | - | - | - | <10.0 | - | - | - | - | - | - | 8.1 | 2.4 ± 0.9 | - | - | - |
| Omicron (BA.1) | 1.2 | 3.1 | 5.4 | 0.6 | <2.4 | <5.0 | 1.4 | - | 3.2 | 10.3 | - | - | - | 5.8 | - | - | 3.0 | - | 2.0 | - | - | 17.3 | 5.1 | 26.2 |
| Delta-Omicron (AY.45-BA.1) | - | - | 35.2 | - | - | - | - | - | - | - | - | - | - | - | - | - | - | - | - | - | - | - | - | - |
| Omicron (BA.1.1) | 2.4 | - | - | 1.8 | - | <5.0 | 1.5 | - | - | 5.7 | - | - | - | 3.9 | - | - | 2.0 | - | - | - | - | - | - | - |
| Omicron (BA.2) | 2.2 | 3.9 | 11.1 | 0.9 | - | 1.0 | 1.1 | - | - | 2.8 | 3.0 | - | - | 3.3 | 6.1  ± 0.7 | - | 2.0 | 1.1 | 4.0 | 3.8 | 1.7 ± 0.8 | - | 0.6 | 11.5 |
| Omicron (BA.2.10.4) | - | - | - | - | - | - | - | - | - | - | - | 3.0 | - | - | - | - | - | - | - | - | - | - | - | - |
| Omicron (BA.2.11) | - | - | - | - | - | - | - | - | - | - | - | - | - | - | - | - | - | - | - | 2.3 | - | - | - | - |
| Omicron (BA.2.13) | - | - | - | 1.0 | - | - | - | - | - | - | - | - | - | - | - | - | - | - | - | - | - | - | - | - |
| Omicron (BA.2.12.1) | - | 3.1 | - | 0.8 | - | 1.0 | - | - | - | 3.1 | - | - | - | 4.0 | - | - | 2.0 | - | - | 5.5 | - | - | - | - |
| Omicron (BA.2.38) | - | - | - | 1.0 -1.3 | - | - | - | - | - | - | - | - | - | - | - | - | - | - | - | - | - | - | - | - |
| Omicron (BA.2.38.1) | - | - | - | 1504.0 | - | - | - | - | - | - | - | - | - | - | - | - | - | - | - | - | - | - | - | - |
| Omicron (BA.2.74) | - | - | - | 2.6 | - | - | - | - | - | - | - | - | - | - | - | - | - | - | - | - | - | - | - | - |
| Omicron (BA.2.75) | - | - | - | 2.2 | - | 7.0 | - | - | - | - | 15.0 | - | - | - | 6.2 ± 2.8 | **-** | - | - | - | - | 34.0 ± 6.9 | - | - | - |
| Omicron (BA.2.75.2) | - | - | - | 3.0 | - | - | - | - | - | - | - | 2.0 | - | - | - | - | - | - | - | - | - | - | - | - |
| Omicron (BA.2.75.4) | - | - | - | 4.0 | - | - | - | - | - | - | - | - | - | - | - | - | - | - | - | - | - | - | - | - |
| Omicron (BA.2.76) | - | - | - | 1.3 | - | - | - | - | - | - | - | - | - | - | - | - | - | - | - | - | - | - | - | - |
| Omicron (BA.2.77) | - | - | - | 0.9 | - | - | - | - | - | - | - | - | - | - | - | - | - | - | - | - | - | - | - | - |
| Omicron (BA.2.79) | - | - | - | 14.0 | - | - | - | - | - | - | - | - | - | - | - | - | - | - | - | - | - | - | - | - |
| Omicron (BA.3) | 1.4 | - | - | 1.1 | - | - | - | - | - | - | - | - | - | - | - | - | - | - | - | - | - | - | - | - |
| Omicron (BA.4) | - | 4.1 | - | 0.8 | - | 1.0 | - | 0.8 | - | 2.2 | - | - | - | 2.9 | - | 12.0 | 2.0 | 1.2 | - | 6.3 | 1.3 ± 0.3 | - | - | - |
| Omicron (BA.4.6) | - | - | - | 1.0 | - | - | - | 1.0 | - | - | - | 2.0 | - | - | - | - | - | 1.2 | - | - | - | - | - | - |
| Omicron (BA.4.7) | - | - | - | - | - | - | - | 1.4 | - | - | - | - | - | - | - | - | - | 1.0 | - | - | - | - | - | - |
| BA.4/5-R346T | - | - | - | - | - | - | - | - | - | - | - | - | - | - | - | - | - | 1.5 | - | - | - | - | - | - |
| BA.4/5-R346S | - | - | - | - | - | - | - | - | - | - | - | - | - | - | - | - | - | 0.7 | - | - | - | - | - | - |
| BA.4/5-N658S | - | - | - | - | - | - | - | - | - | - | - | - | - | - | - | - | - | 0.6 | - | - | - | - | - | - |
| Omicron (BA.5) | - | - | - | 0.9 | - | - | - | 0.8 | - | - | 7.0 | 1.0 | - | 3.3 | 2.4 ± 1.3 | 15.0 | - | - | - | - | - | - | - | - |
| Omicron (BA.5.1.12) | - | - | - | 98.0 | - | - | - | - | - | - | - | - | - | - | - | - | - | - | - | - | - | - | - | - |
| Omicron (BA.5.2.7) | - | - | - | 2108.0 | - | - | - | - | - | - | - | - | - | - | - | - | - | - | - | - | - | - | - | - |
| Omicron (BA.5.5.1) | - | - | - | 5.1 | - | - | - | - | - | - | - | - | - | - | - | - | - | - | - | - | - | - | - | - |
| Omicron (BA.5.6.2) | - | - | - | 1662.0 | - | - | - | - | - | - | - | - | - | - | - | - | - | - | - | - | - | - | - | - |
| Omicron (BA.5.9) | - | - | - | - | - | - | - | 1.3 | - | - | - | - | - | - | - | - | - | 0.9 | - | - | - | - | - | - |
| Omicron (BF.16) | - | - | - | 2.7 | - | - | - | - | - | - | - | - | - | - | - | - | - | - | - | - | - | - | - | - |
| Omicron (BL.1) | - | - | - | 2.8 | - | - | - | - | - | - | - | - | - | - | - | - | - | - | - | - | - | - | - | - |

IC_50_ (ng/ml) was the measure of neutralization activity, unless otherwise stated.

^‡^ Fold change in IC_50_ values (µg/ml) compared to wild-type.

ᶲ IC_99_ (ng/ml).
